# Incidence and risk factors of acute kidney injury in critically ill patients from a single centre in Brazil: a retrospective cohort analysis

**DOI:** 10.1038/s41598-019-54674-1

**Published:** 2019-12-02

**Authors:** Reginaldo Passoni dos Santos, Ariana Rodrigues da Silva Carvalho, Luis Alberto Batista Peres

**Affiliations:** 1 Postgraduate Program in Biosciences and Health, Western Parana State University, Cascavel, Brazil; 2 Department of Nursing, Western Parana State University, Cascavel, Brazil; 3 Department of Medicine, Nephrology Division, Western Parana State University, Cascavel, Brazil

**Keywords:** Acid, base, fluid, electrolyte disorders, Acute kidney injury

## Abstract

Studies with a comprehensive analysis of the epidemiology of acute kidney injury (AKI) in intensive care units (ICUs) are still limited in developing countries. The aim of this study is to identify the incidence and risk factors of AKI in critically ill patients from a Brazilian ICU. We performed a retrospective analysis of the records of patients admitted to a single-centre adult ICU in Brazil between 1 January 2011 and 31 December 2016. The KDIGO criteria were used to define AKI. Univariate and multivariate data analyses were carried out. We included 1,500 patients. The incidence of AKI was 40.5%, and the AKI dialysis rate was 13%. The predictors of AKI at ICU admission included hypertension [odds ratio (OR) = 1.44, p 0.017], high serum creatinine concentration [OR = 3.54; p < 0.001], low serum albumin concentration [OR = 1.42, p 0.015], high APACHE II score [OR = 2.10; p < 0.001] and high SAPS 3 [OR = 1.75; p < 0.001]. The incidence of AKI was high, and we identified the predictors of AKI among critically ill Brazilian patients. The results of this study may contribute to the implementation of targeted therapies.

## Introduction

Acute kidney injury (AKI) is a frequent and serious complication among critically ill patients in intensive care units (ICUs) and has short- and long-term consequences^1,2^. Socioeconomic differences between countries reflect differences in the epidemiology of this event^3–5^. However, studies with comprehensive analyses of the epidemiology and predictors of AKI in ICUs are still limited, especially in developing countries^6^.

Studies performed in developing countries contribute to the recognition of the epidemiological panorama of AKI in these regions and to the establishment of guidelines and targeted therapies^7^. In a prospective cohort study performed in 22 ICUs in mainland China, the incidence of AKI was 31.6%, and the stage of the event was well correlated with clinical deterioration and patient mortality^8^. In two other large studies, it was found that high serum chloride concentrations^9^ and microalbuminuria^10^ were well correlated with the development of AKI in nonselected and septic patients, respectively. This evidence from large scientific studies grounds the direction of assistance resources, which in developing countries, are very limited and need to be used on a cost-effective basis.

In Brazil, the epidemiology data for AKI in ICUs are still limited. In recent years, only two studies^11,12^ have been published, and these include nonrobust numbers of patients in the analyses, which makes it difficult to understand the national epidemiological panorama of AKI and to establish a consensus of care. In an attempt to minimise the financial cost and logistical difficulties of carrying out multicentre studies, Zampieri *et al*.^13^ encouraged Brazilian intensivists to participate in a cloud-based national registry of adult ICU patients in Brazil. However, at the moment, no epidemiology data on AKI in ICUs have been published from this database, and thus, single-centre studies remain important for recognising the epidemiological burden of AKI in Brazilian ICUs. Therefore, the aim of this study is to identify the incidence of and risk factors for AKI in critically ill patients in a Brazilian ICU.

## Methods

### Design, setting and population of the study

A retrospective cohort analysis was performed in the adult ICU of a public teaching hospital located in the southern region of Brazil. All patients aged 18 years or older, as well as those who stayed in the ICU for 48 hours or longer, were included in the study. The following patients were excluded: those with a medical registry of AKI before ICU admission (ICU-ad); those transferred to the ICU for renal replacement therapy (RRT); those with serum creatinine (sCr) levels at ICU-ad greater than 4.0 mg/dL; those diagnosed with a chronic kidney disease; those undergoing a kidney transplant; and those with no paper records. For patients with multiple admissions to the ICU, the admission with the longest stay was considered for the analysis. The reporting of this study follows the STROBE (Strengthening the Reporting of Observational Studies in Epidemiology) checklist^14^.

### Data collection

The data of patients admitted to the ICU between 1 January 2011 and 31 December 2016 were collected. The data were retrieved between October 2016 and January 2018 from the patients’ electronic and paper records. To extract the information of interest, a data collection instrument, which was created specifically for this study, previously validated (content and face validity) and applied in the pilot study, was used.

All data were collected at the time of ICU admission and included the patients’ baseline characteristics (age, sex and race), comorbidities (hypertension, diabetes, cardiovascular disease, cancer, human immunodeficiency virus and other comorbidities), in-hospital admission motive (surgical or clinical); in-hospital location before ICU-ad (emergency department, surgical centre, ward or another hospital); ICU-ad conditions (need for mechanical ventilation, nosocomial infection, sepsis, shock or polytrauma), urinary parameters (urine output in the first 24 hours and oliguria at ICU-ad) and length of stay (in the hospital and in the ICU).

After retrieving the ICU-ad records, the prognostic Acute Physiology and Chronic Health Evaluation (APACHE) II score^15^, the Simplified Acute Physiology Score (SAPS) 3 score^16^ and the Sequential Organ Failure Assessment (SOFA) score^17^ were calculated.

Data about the haemodynamic parameters (heart rate, respiratory rate, blood temperature, systolic and diastolic blood pressure, mean arterial pressure, central venous pressure and Glasgow coma scale) and laboratory parameters (serum creatinine, urea, sodium, potassium, chloride, lactate and albumin concentrations) were also retrieved.

Additionally, the following biochemical imbalances were verified at ICU-ad: hyponatraemia (serum concentration of sodium <135 mEq/L); hypernatraemia (serum concentration of sodium >145 mEq/L); hypokalaemia (serum concentration of potassium < 3.5 mEq/L); hyperkalaemia (serum concentration of potassium >5.5 mEq/L); hypochloraemia (serum concentration of chloride <97 mEq/L); hypochloraemia (serum concentration of chloride >107 mEq/L); hypoalbuminaemia (serum concentration of albumin <3.5 g/dL) and metabolic acidosis (arterial blood pH <7.35 and serum concentration of bicarbonate <22 mEq/L).

### Criteria for an AKI diagnosis

AKI was defined and classified by the Kidney Disease: Improving Global Outcomes (KDIGO) guidelines, which use serum creatinine and urinary volume values for the AKI diagnosis^18^. To identify the cases of AKI, both KDIGO criteria (worst sCr and urine output) were considered. To apply the KDIGO criteria, we considered the lowest sCr at ICU-ad as the baseline value; however, to identify cases of AKI at ICU-ad, we considered the worst pre-ICU-ad as the baseline creatinine. For patients with AKI, the need for RRT was verified.

### Outcomes

The verified outcomes were the incidence of AKI and need for RRT, as well as the ICU mortality rate (overall, for patients with AKI and for patients with a need for RRT).

### Statistical analysis

The absolute and relative incidences of AKI and need for RRT were calculated. To identify the risk factors, the patients were divided into two groups (with and without AKI), and comparative analyses were performed. The normality (using the Shapiro-Wilk test) and homoscedasticity (using test F) of the data were analysed; the categorical variables are presented as the absolute frequency (percentage), and the continuous variables as presented the means ± standard deviations (SD) and minimum/maximum values or as medians and interquartile ranges (IQR 25–75%).

Comparisons among categorical variables were performed using the chi-square or Fisher’s exact test, and those among the continuous variables were performed using the Student’s t-test or the Mann–Whitney U test, depending on which was appropriate. The variables that presented p-values less than 0.25 were selected and included in the multivariate logistic regression^19^.

To avoid confounders due to multicollinearity problems in the final regression model, the variance inflation factor (VIF) indicator was analysed. A VIF greater than 5 indicated multicollinearity problems, and these variables were excluded from the final adjusted model.

The model calibration was assessed using the Hosmer-Lemeshow test, and the discrimination was assessed by the area under the receiver operating characteristic curve (AUROC). The AUROC was also applied to determine the cut-off points of the quantitative variables maintained in the final multivariate regression model. A p-value less than 0.05 was considered statistically significant, with a 95% confidence interval (CI). All analyses were performed using R statistical software (R Core Team, version 3.4.3, Vienna, Austria).

### Ethical approval

The study was approved by the Ethics Committee of the Western Paraná State University (approval number: 1.622.962) and authorised by the general directorate of the Western Parana University Hospital. The requirement for informed consent was waived. This study was conducted in accordance with the Brazilian National Health Council and the Declaration of Helsinki.

## Results

**Figure 1:**
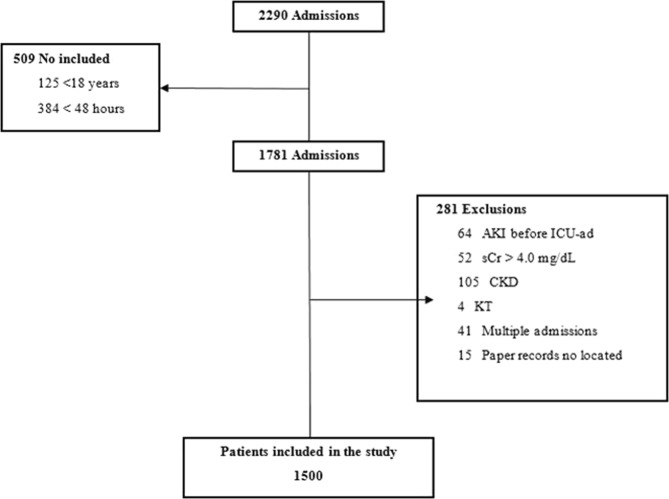
Flow chart of the patient selection process. Note: *AKI* acute kidney injury, *CKD* chronic kidney disease, *KT* kidney transplant, *sCr* serum creatinine.

During the study period, 2,290 patients were admitted to the ICU, and of these, 1,500 were included in the final analysis (Fig. 1). Of the 1,500 patients included, 608 (40.5%) had AKI, and according to KDIGO staging criteria, 35.5% (n = 216) of the total AKI patients had stage 1 AKI, 25.7% (n = 156) had stage 2 AKI, and 38.8% (n = 236) had stage 3 AKI. Of all AKI patients, 13% (n = 79) required RRT.

The median age of patients with and without AKI was 53 (IQR 38–67) and 42 (IQR 29–58) years, respectively (p < 0.001). All comorbidities analysed were found more frequently in the AKI patients than in those without AKI (p < 0.05). Patients with AKI had more nosocomial infections (n = 159 (26.2%) vs n = 141 (15.8%); p < 0.001) and sepsis (n = 62 (10.2%) vs n = 53 (6.0%); p 0.004) at ICU-ad than those without AKI. The median urine output in the first 24 hours at ICU was 2.4 (IQR 1.5–3.4) litres for patients with AKI and 2.9 (IQR 2.1–4.0) litres for patients without AKI. AKI patients also had higher APACHE II, SAPS 3 and SOFA scores than those without AKI (Table 1).

**Table 1:** The general characteristics of the patients with and without AKI.

Variables	No AKI (*n* = 892)	AKI (*n* = 608)	*p*
**Baseline characteristics**			
Age (years)	42 (29–58)	53 (38–67)	<0.001
Sex (male)	566 (63.5%)	371 (61.0%)	0.353
Race (Caucasian)	752 (84.4%)	520 (85.5%)	0.600
**Comorbidities**			
Hypertension	215 (24.1%)	247 (40.6%)	<0.001
Diabetes	73 (8.2%)	98 (16.1%)	<0.001
Cardiovascular disease	69 (7.7%)	92 (15.1%)	<0.001
Cancer	127 (14.3%)	50 (8.2%)	0.001
Human Immunodeficiency Virus	19 (2.1%)	27 (4.4%)	0.017
Others	212 (23.8%)	201 (33.1%)	<0.001
In-hospital admission motive			<0.001
Surgical	654 (73.4%)	361 (59.4%)	
Clinical	237 (26.6%)	247 (40.6%)	
In-hospital location before ICU-ad			0.002
Emergency department	488 (54.8%)	360 (59.2%)	
Surgical centre	336 (37.7%)	182 (29.9%)	
Ward	56 (6.3%)	61 (10.0%)	
Other hospital	11 (1.2%)	5 (0.8%)	
ICU-ad motive			<0.001
Surgical	629 (70.7%)	354 (58.2%)	
Clinical	261 (29.3%)	254 (41.8%)	
Clinical conditions at ICU-ad^*^			
Mechanical ventilation	753 (84.5%)	537 (88.3%)	0.044
Nosocomial infection	141 (15.8%)	159 (26.2%)	<0.001
Sepsis	53 (6.0%)	62 (10.2%)	0.004
Shock	138 (12.7%)	51 (12.4%)	0.946
Polytrauma	367 (41.2%)	160 (26.3%)	<0.001
Urine output (first 24 hours, litres)	2.9 (2.1–4.0)	2.4 (1.5–3.4)	<0.001
**Mortality prognoses in the first 24 hours (points)**			
APACHE II	20 (16–24)	26 (21–30)	<0.001
SAPS 3	62 ± 15 (22–107)	73 ± (26–120)	<0.001
SOFA	8 (5–10)	10 (7–12)	<0.001
**Outcomes (length of stay, days)**			
Hospital	20 (13–31)	23 (12–39)	0.002
ICU	7 (4–13)	12 (6–22.5)	<0.001

Continuous variables are presented as medians [interquartile range], except for the SAPS 3, which are presented as the mean ± standard derivation (minimum – maximum), and categorical variables are presented as numbers (%). *ICU-ad* Intensive care unit admission; *APACHE II* Second version of the Acute Physiologic and Chronic Health Evaluation*; SAPS 3* Third version of the Simplified Acute Physiology score*; SOFA* Sequential Organ Failure Assessment.

*The clinical conditions are not mutually exclusive; it is possible for the same patient to have more than one clinical condition at ICU-ad.

The median central venous pressure at ICU-ad was 11.0 (IQR 7.3–14.7) and 9.5 (IQR 6.6–12.5) mmHg in patients with and without AKI, respectively. The median sCr was greater in AKI patients than in those without AKI (0.85 (IQR 0.69–1.06) vs 1.11 (IQR 0.78–1.69); p < 0.001), and AKI patients also had more hyperkalaemia (n = 39 (6.4%) vs n = 19 (2.1%); p < 0.001), hypoalbuminaemia (n = 575 (94.6%) vs n = 783 (87.9%); p < 0.001) and metabolic acidosis (n = 326 (53.6% vs 337 (37.8%); p < 0.001) at ICU-ad than those without AKI (Table 2).

**Table 2:** Haemodynamic, laboratory parameters, and biochemical imbalances in the patients with and without AKI.

Variables	No AKI (*n = *892)	AKI (*n = *608)	*p*
**Haemodynamic parameters**			
Heart rate (bpm)	88 (73–106)	93 (75–111)	0.006
Respiratory rate (ipm)	18 (18–20)	18 (18–21)	0.011
Blood temperature (°C)	36.3 (35.5–37.0)	36.1 (35.3–36.9)	0.029
Systolic arterial pressure (mmHg)	121 (109–140)	120 (100–140)	0.002
Diastolic arterial pressure (mmHg)	70 (60–80)	70 (60–80)	0.060
Mean arterial pressure (mmHg)	89 (77–102)	87 (74–99)	0.008
Central venous pressure (mmHg)	9.5 (6.6–12.5)	11.0 (7.3–14.7)	<0.001
Glasgow Coma Scale (points)	3 (3–8)	3 (3–4)	<0.001
Creatinine (mg/dL)	0.85 (0.69–1.06)	1.10 (0.78–1.69)	<0.001
Urea (mg/dL)	27 (19–37)	40 (26–66)	<0.001
Sodium (mEq/L)	137 (134–139)	137 (133–1140)	0.784
Potassium (mEq/L)	3.9 (3.6–4.3)	4.1 (3.6–4.6)	<0.001
Chloride (mEq/L)	110 (107–114)	111 (107–116)	0.013
Lactate (mmol/L)	1.60 (1.10–2.50)	1.80 (1.30–2.60)	0.005
Albumin (g/dL)	2.60 (2.20–3.20)	2.40 (2.00–2.80)	<0.001
**Biochemical imbalances**			
Hyponatremia	250 (28.1%)	207 (34.0%)	0.016
Hypernatremia	33 (3.7%)	36 (5.9%)	0.059
Hypokalaemia	172 (19.3%)	123 (20.2%)	0.706
Hyperkalaemia	19 (2.1%)	39 (6.4%)	<0.001
Hypochloraemia	23 (2.6%)	16 (2.6%)	1.000
Hyperchloremia	619 (69.5%)	426 (70.1%)	0.851
Hypoalbuminemia	783 (87.9%)	575 (94.6%)	<0.001
Metabolic acidosis	337 (37.8%)	326 (53.6%)	<0.001

Continuous variables are presented as medians [interquartile range], and categorical variables are presented as numbers (%).

*ICU-ad* Intensive care unit admission *AKI* Acute kidney injury.

As seen in Table 3, we identified the risk factors for AKI at ICU-ad with a logistic regression. According to the multivariate analysis, the independent risk factors for AKI were a history of hypertension (OR 1.44, 95% CI 1.07–1.94; p 0.017), serum creatinine concentration > 1.16 mg/dL (OR 3.54, 95% CI 2.65–4.73; p < 0.001), serum albumin concentration ≤ 2.81 g/dL, APACHE II score > 25 points (OR 2.10, 95% CI 1.56–2.81; p < 0.001) and SAPS 3 score > 68 points (OR 1.75, 95% CI 1.31–2.33; p < 0.001).

**Table 3:** Logistic regression analysis to identify the risk factors for AKI at ICU-ad in critically ill Brazilian patients.

Variables	Univariate analysis		Multivariate analysis	
	OR (95% CI)	*p*	OR (95% CI)	*p*
Age	1.01 (0.99–1.02)	0.309	—	—
**Comorbidities**				
Hypertension	1.28 (0.86–1.92)	0.223	1.44 (1.07–1.94)	0.017
Diabetes	1.11 (0.66–1.85)	0.701	—	—
Cardiovascular disease	1.36 (0.82–2.26)	0.234	—	—
Human immunodeficiency virus	1.89 (0.81–4.39)	0.139	—	—
Stay in ward before ICU-ad	1.05 (0.57–1.93)	0.886	—	—
**Vital signs and haemodynamic parameters**				
Respiratory rate	1.02 (0.98–1.05)	0.319	—	—
Cardiac rate	1.00 (0.99–1.01)	0.709	—	—
Systolic arterial pressure	0.99 (0.98–1.00)	0.002	0.80 (0.59–1.08)	0.151
Diastolic arterial pressure	1.02 (1.00–1.03)	0.009	1.15 (0.84–1.56)	0.385
Central venous pressure	1.00 (0.97–1.03)	0.989	—	—
**Clinical conditions**				
Invasive mechanical ventilation	0.67 (0.29–1.55)	0.346	—	—
Nosocomial infection	0.77 (0.50–1.18)	0.227	—	—
Sepsis	0.71 (0.38–1.32)	0.278	—	—
Shock	1.12 (0.69–1.80)	0.649	—	—
Urine output (first 24 hours in ICU)	0.96 (0.44–2.12)	0.381	—	—
**Laboratory parameters (serum values)**				
Creatinine (>1.16 mg/dL)	3.07 (1.95–4.85)	<0.001	3.54 (2.65–4.73)	<0.001
Urea	1.01 (1.00–1.01)	0.116	—	—
Potassium	0.96 (0.69–1.34)	0.809	—	—
Lactate	1.00 (0.91–1.10)	0.985	—	—
Albumin (≤2.81 g/dL)	0.78 (0.56–1.09)	0.140	1.42 (1.07–1.89)	0.015
Aspartate alanine transferase	1.10 (1.01–1.20)	0.037	1.24 (0.92–1.66)	0.157
**Biochemical imbalance**				
Hyponatremia	1.41 (0.96–2.06)	0.078	—	—
Hypernatremia	1.39 (0.71–2.73)	0.336	—	—
Hypokalaemia	1.20 (0.72–1.98)	0.483	—	—
Hyperkalaemia	0.79 (0.28–2.21)	0.658	—	—
Hypochloraemia	0.86 (0.32–2.34)	0.769	—	—
Hyperchloremia	0.91 (0.59–1.41)	0.689	—	—
Hypoglycaemia	1.11 (0.25–5.05)	0.888	—	—
Hyperglycaemia	0.74 (0.48–1.13)	0.162	—	—
Hypoalbuminemia	1.11 (0.58–2.13)	0.743	—	—
Metabolic acidosis	0.92 (0.60–1.41)	0.692	—	—
**Prognostic scores in the first 24 hours**				
APACHE 2 (>24 points)	1.06 (1.01–1.10)	0.010	2.10 (1.56–2.81)	<0.001
SAPS 3 (>68 points)	1.01 (1.00–1.03)	0.122	1.75 (1.31–2.33)	<0.001
SOFA	1.00 (0.92–1.10)	0.943	—	—

Performance measures of the final multivariate model: The calibration was assessed by the Hosmer-Lemeshow goodness of fit test, χ^2^ 9.930, df = 8, p-value = 0.270. Discrimination was assessed by the area under the receiver operating characteristic curve for the occurrence of AKI = 0.80 (95% CI 0.78–0.83). Patients included in the analysis, *n = *1,389.

*OR* Odds ratio *CI* Confidence internal *AKI* Acute kidney injury *ICU-ad* Intensive care unit admission *APACHE II* Second version of the Acute Physiologic and Chronic Health Evaluation*; SAPS 3* Third version of the Simplified Acute Physiology Score*; SOFA* Sequential Organ Failure Assessment.

The median length of hospital stay for AKI patients was 23 days (IQR 12–39 days) and that for patients without AKI was 20 days (IQR 13–31 days) (p 0.002). AKI patients also had longer ICU stays than those without AKI (median = 12 days, IQR 6–22 days vs median = 7, IQR 4–13 days; p < 0.001). The overall mortality rate was 18.5% (n = 277), while among AKI patients and AKI dialysis patients, the mortality rates was 39.1% (n = 238) and 62.0% (n = 49), respectively.

## Discussion

A comprehensive analysis of the epidemiology of AKI was carried out and evaluated a large number of predictors in a population of critically ill Brazilian patients. In recent years, few studies on the epidemiology of AKI based on such a comprehensive analysis as that in this study have been performed. The incidence of AKI according to the KDIDGO criteria was 40.5%. In the southeast region of country, the incidence of AKI according to the AKIN (Acute Kidney Injury Network) criteria^20^ was 25.5%^11^, and in another study previously conducted in the same region, the incidence was 53.2%;^12^ however, the previous study analysed only 152 patients and used the RIFLE (Risk, Injury, Failure, Loss, End-Stage) criteria^21^.

The incidence of AKI in this study is comparable to that found in other large studies. In Finland, in a multicentre study with 2,901 critically ill patients, Nisula *et al*.^22^ verified that the incidence of AKI according to the KDIGO criteria was 39.3%. Wen *et al*.^8^ analysed data from 3,063 critically ill Chinese patients and verified that 31.6% of the patients had AKI using the RIFLE criteria. In a single-centre prospective study, Halle *et al*.^23^ analysed 2,402 critically ill African patients, and the overall incidence of AKI according to the KDIGO criteria was 22.3%.

Recently, Hoste *et al*.^4^ published the results of a multinational study on the epidemiology of AKI among 1,802 patients from 97 ICUs around the world, and the overall incidence was 57.3%. In another international multicentre study, Bouchard *et al*.^3^ identified that 19.2% of the 6,647 patients followed during the first seven days after ICU-ad had AKI and that the incidence of AKI in patients from developing and developed countries was 19.9% and 19.2%, respectively.

Several factors influence the incidence of AKI in ICUs, including lack of the standardised criteria for AKI diagnosis, limited medical resources for AKI management and patient profile^3–5,24,25^. A national cross-sectional survey carried out in 36 Brazilian hospitals investigated AKI management practices by intensivists and found that 47.1% did not apply standardised criteria to identify AKI in their patients^26^. An international survey identified the existence of almost 100 different criteria to diagnose AKI that are applied in daily clinical practice in ICUs around the world^27^.

As for resources, difficulties in each country regarding the availability of health resources contribute to the increase in the incidence of AKI^3,5,7,24^. In this study, the incidence of AKI was evaluated in a general adult population from a mixed ICU. Subgroups of critically ill patients, such as those with sepsis and those who underwent major surgery, had a higher incidence of AKI than mildly ill patients^25^.

Among AKI patients, the RRT rate was 13% in our study. Other studies reported RRT rates of 7.3%^23^, 10.2%^22^ and 36.5%^8^. The need for RRT is one of the main complications of AKI and has a strong association with failed recovery of renal function and a high mortality rate; many aspects of RRT have sparked controversy^28^.

The identification of patients at risk and the early recognition of AKI are essential for the implementation of responses capable of promoting adequate renal support and the promotion of rehabilitation^29^. In this study, the a high sCr value (>1.16 mg/dL) at ICU-ad was an independent risk factor for AKI.

In other Brazilian studies, a high sCr value at ICU-ad was also a predictor of AKI^11,12^. After critically assessing patients with complicated intra-abdominal infections, Suarez de la Rica *et al*.^30^ found that the sCr level at ICU-ad was an AKI predictor. After analysing data from 3,107 critically Chinese patients, Luo *et al*.^31^ verified that small increases in the sCr level at ICU-ad reflected increases in the incidence of AKI. Federspiel *et al*.^32^ evaluated AKI duration among patients with acute respiratory distress syndrome and verified that high sCr levels at ICU-ad were associated with persistent AKI. In addition, for AKI patients, higher sCr levels at ICU-ad seem to be associated with a greater need for RRT and a higher mortality rate^2,25^.

In this study, APACHE II scores (>24 points) and SAPS 3 scores (>68 points) at ICU-ad were also AKI risk factors. Elevated scores of these prognostic mortality systems were also reported by other authors^3,4,8,11^, indicating that these systems may be useful in the prediction of not only AKI but also mortality.

A randomised controlled trial reported that patients with serum albumin levels lower than 3.0 g/dL had a greater incidence of AKI^33^. Takaya *et al*.^34^ observed that a small reduction (≥0.3 g/L) in serum albumin concentration at ICU-ad was independently associated with AKI occurrence in patients with acute decompensated heart failure. In a meta-analysis of observation studies, the cut-offs for defining hypoalbuminemia varied, but low serum albumin concentrations were clearly identified as an independent risk factor for AKI^35^. In this study, serum albumin concentrations ≤ 2.81 g/dL was a risk factor for AKI.

Many causes of AKI are preventable and treatable^1^, but inadequate or delayed approaches may result in serious negative outcomes^2^. In this sense, it was verified that AKI patients had a longer median length of stay than patients without AKI (in the hospital: 23 vs 20 days, p 0.002; in the ICU: 12 vs 7 days, p < 0.001). Comprehensive international studies show that, around the world, patients with AKI had a longer length of stay than those without AKI and that this was among the main reasons that these patients had a greater need for care^3–5^.

The mortality rate of AKI patients was 39.1%, which is higher than that observed in other studies from Brazil^11,12^ and from other countries^8,22^. In this study, the mortality risk factors were not verified, but other authors reported that the main mortality risk factors in AKI patients included length of ICU stay, need for RRT, elderly age, need for invasive mechanical ventilation, hypernatraemia, infections and neoplasms^11,12,36^.

Finally, it should be emphasised that this study has some limitations, including the methodological design (retrospective cohort), local comprehensiveness (single-centre), lack of evaluations of the duration of AKI during the patients’ ICU stay, relationship between risk factors and mortality, and rate of renal recovery in AKI patients, and a lack of data on long-term AKI consequences.

We suggest that these limitations be addressed in future research. Future research may include the utilisation of a new AKI biomarker to improve the early recognition of the disease and advances in RRT management, which may reduce the impact of AKI in critically ill patients.

## Conclusion

The incidence of AKI is high, and the data are consistent with the literature. It was identified that the predictors of AKI among critically ill patients from a single-centre Brazilian ICU at admission included a history of hypertension, high serum creatinine and low serum albumin concentrations, and high APACHE II and SAPS 3 scores. However, these findings need to be confirmed by more studies, especially multicentre studies with a prospective design.

### Highlights

- Critically ill Brazilian patients present a high incidence of AKI.
- A high serum creatinine concentration (>1.16 mg/dL) at ICU admission is the main risk factor for AKI.
- Hypoalbuminemia is also a predictor of AKI.
- Applying prognostic indexes at ICU admission may help in the early diagnosis of AKI.
- Preventing and treating reversible cases of AKI can avoid the progression of AKI to chronic kidney disease.

## Data Availability

All data for this research are available in this manuscript.
